# Case report: EBV-related eye orbits and sinuses lymphohistiocytic infiltration responsive to rituximab in a patient with X lymphoproliferative syndrome type 1

**DOI:** 10.3389/fimmu.2024.1370991

**Published:** 2024-04-03

**Authors:** Giuliana Giardino, Vittoria Lanni, Massimo Mascolo, Daniela Russo, Emilia Cirillo, Roberta Romano, Francesca Cillo, Laura Grilli, Maria Rosaria Prencipe, Adriana Iuliano, Giovanni Uccello, Carmela De Fusco, Giuseppe Menna, Giulia Scalia, Giuseppe Portella, Claudio Pignata

**Affiliations:** ^1^ Department of Translational Medical Sciences, Pediatric Section, Federico II University of Naples, Naples, Italy; ^2^ Department of Neurosciences, Reproductive Sciences and Dentistry, University of Naples Federico II, Naples, Italy; ^3^ Department of Advanced Biomedical Sciences, Pathology Unit, Federico II University of Naples, Naples, Italy; ^4^ Pediatric Hematology and Oncology, Pausilipon Hospital, Naples, Italy; ^5^ Clinical and Experimental Cytometry Unit, Centre for Advanced Biotechnology Franco Salvatore, CEINGE, Naples, Italy; ^6^ Department of Translational Medical Sciences, Virology Section, Federico II University of Naples, Naples, Italy

**Keywords:** X lymphoproliferative syndrome type 1, eye lymphohistiocytic infiltration, EBV, rituximab, *SH2D1A*

## Abstract

**Background and aims:**

X lymphoproliferative syndrome type 1 (XLP1) is a rare inborn error of immunity due to mutations of *SH2D1A*, encoding for slam-associated protein (SAP). The clinical phenotype includes severe mononucleosis, hemophagocytic lymphohistiocytosis (HLH), and B-cell lymphomas.

**Methods:**

We report the case of a child affected with XLP1 who presented with an incomplete HLH, triggered by Epstein–Barr virus (EBV) and treated with rituximab, involving orbits and paranasal sinuses.

**Results:**

The lesion was indistinguishable from lymphoma, complicating diagnosis and treatment. In addition, considering the high incidence of lymphoma in patients with XLP1, histology helped define its nature, driving therapeutic choices.

**Conclusion:**

We described an unusual presentation of incomplete HLH in a patient affected with XLP1: an EBV-driven infiltration of the orbits and paranasal sinuses. This led us to a challenging differential diagnosis of lymphoma-associated hemophagocytic syndrome, which can be frequently observed in patients with XLP1. Considering the extremely poor prognosis of this clinical finding, we sought for a prompt diagnosis and managed to obtain it and to immediately establish the right treatment on the basis of the pathological finding.

## Introduction

X lymphoproliferative syndrome type 1 (XLP1) is a rare inborn error of immunity due to genetic alterations of the *SH2D1A* gene, encoding for slam-associated protein (SAP) (1). Clinical presentation may be variable, even in patients from the same family (2). The main clinical features include severe mononucleosis, hemophagocytic lymphohistiocytosis (HLH), B-cell lymphomas, and hypogammaglobulinemia. The diagnosis of HLH is based on the HLH-94 criteria and can be confirmed in the presence of five out of eight criteria (3). Atypical or incomplete HLH has been identified in 17% of the familial HLH cases in the German cohort (4). Here, we report the case of a 4.5-year-old child affected with XLP1, presenting with a severe involvement of the orbits and paranasal sinuses in the context of an incomplete HLH, triggered by Epstein–Barr virus (EBV). We will focus on the pitfalls of the diagnostic and therapeutic approach.

## Clinical case

**Figure 1:**
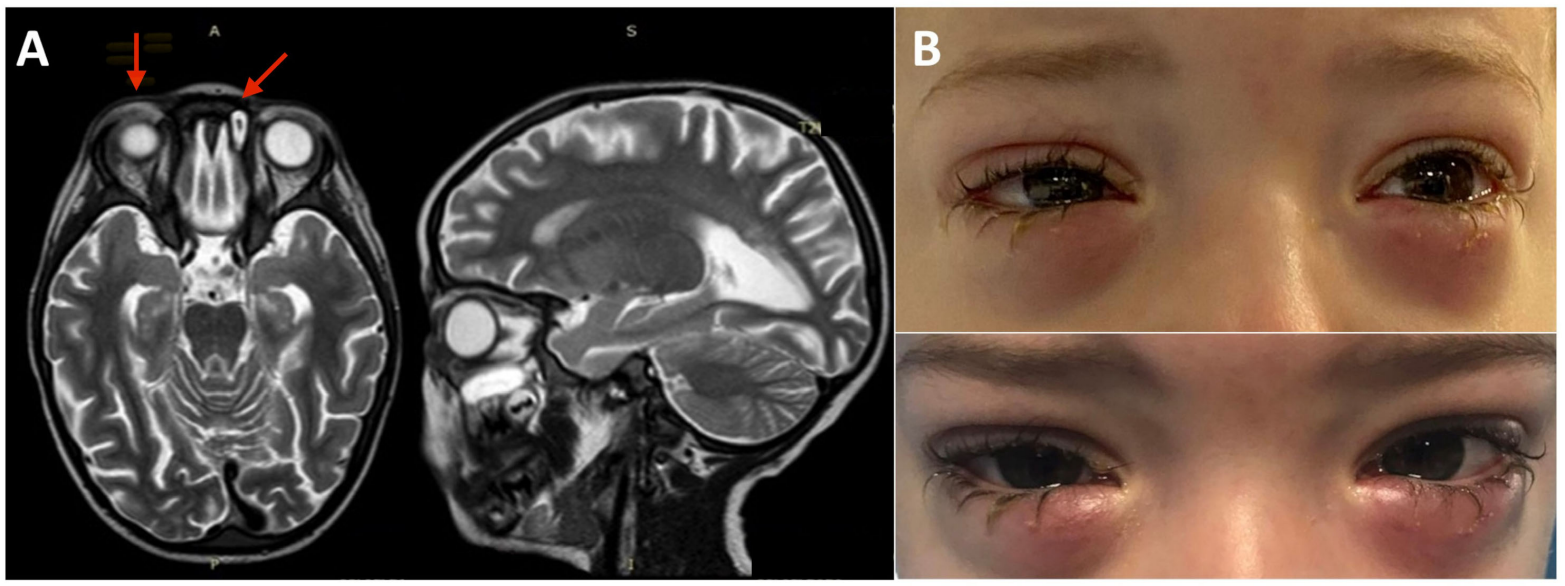
Brain, eye orbit, and paranasal sinus MRI and bilateral lower eyelid edema. **(A)** MRI of brain, eye orbits, and paranasal sinuses showing bilateral obliteration of the ethmoid-maxillary sinuses, an inflammatory thickening of the periorbital subcutaneous adipose tissue, hypointense in T2 in both orbits, but more evident in the right orbit and brain atrophy and fuzzy T2 hyperintensity of the peritrigonal white matter and corpus callosum. **(B)** Bilateral lower eyelid edema at admission (top panel) and during the following weeks before rituximab treatment (bottom panel).

The patient was born at term to healthy non-consanguineous parents, from an uneventful pregnancy. Family history revealed an older brother who died at the age of 2 years of acute EBV-related fulminant liver failure. Clinical history was negative for recurrent or severe infections in the first few years of life. At the age of 4.5 years, he was admitted to our pediatric hospital for fever. Physical examination showed hepatomegaly, splenomegaly, latero-cervical lymphadenopathy, bilateral lower eyelid edema and redness, and nasal congestion. Blood tests revealed anemia (Hb 7.4 g/dL), hyperferritinemia (2,120 ng/mL), hypoalbuminemia (2.6 g/dL), hypertransaminasemia (AST 50 U/L and ALT 85 U/L), increased GGT (76 U/L), LDH (325 U/L) triglycerides (255 mg/dL), and a slight increase of acute phase reactants (CRP and PCT). High-titer EBV-DNA was detected in plasma through polymerase chain reaction (43,000 IU/mL). The study of the bone marrow revealed reduced representation of the erythroid and megakaryocytic series and increased histiocytes in the absence of clonality or hemophagocytosis. Chest x-ray showed an area of consolidation in the middle and basal left lung. Whole-body computed tomography revealed pleural and perihepatic effusion and echocardiography revealed pericardial effusion. XLP was suspected based on the family history. Direct Sanger sequencing failed to amplify the *SH2D1A* gene, and CGH-Array revealed the presence of a 120-kb deletion containing exons 2–4 of SH2D1A in the Xq25 region inherited from the mother. The deletion, predicted to be associated with truncated SH2D1A protein expression, was previously reported by Sumegi et al. in seven patients, coming from two different kindreds. By comparing this mutation with mutations of different types (missense, nonsense, and truncating) or localizations, no significant differences were observed in phenotypes or in severity of disease (5). Direct sequencing also excluded *BIRC4* mutations, responsible for XLP2. Immunological examinations revealed lymphocytosis, B-cell lymphopenia (CD20 + 2%), mild hypogammaglobulinemia (IgG 414 mg/dL, IgA 15.3 mg/dL, and IgM 22.3 mg/dL) and increased activated CD3+HLADR+ (28%). Natural killer cell function test and cytotoxic T-cell degranulation assay results showed abnormality while flow cytometry revealed preserved perforin expression. Brain imaging was performed to exclude neurological involvement, revealing atrophy and fuzzy T2 and FLAIR hyperintensity of the peritrigonal white matter and corpus callosum (**Figure 1A**). Cerebrospinal fluid (CSF) evaluation revealed the presence of few CD3+ cells while EBV-DNA was not detected.

**Figure 2:**
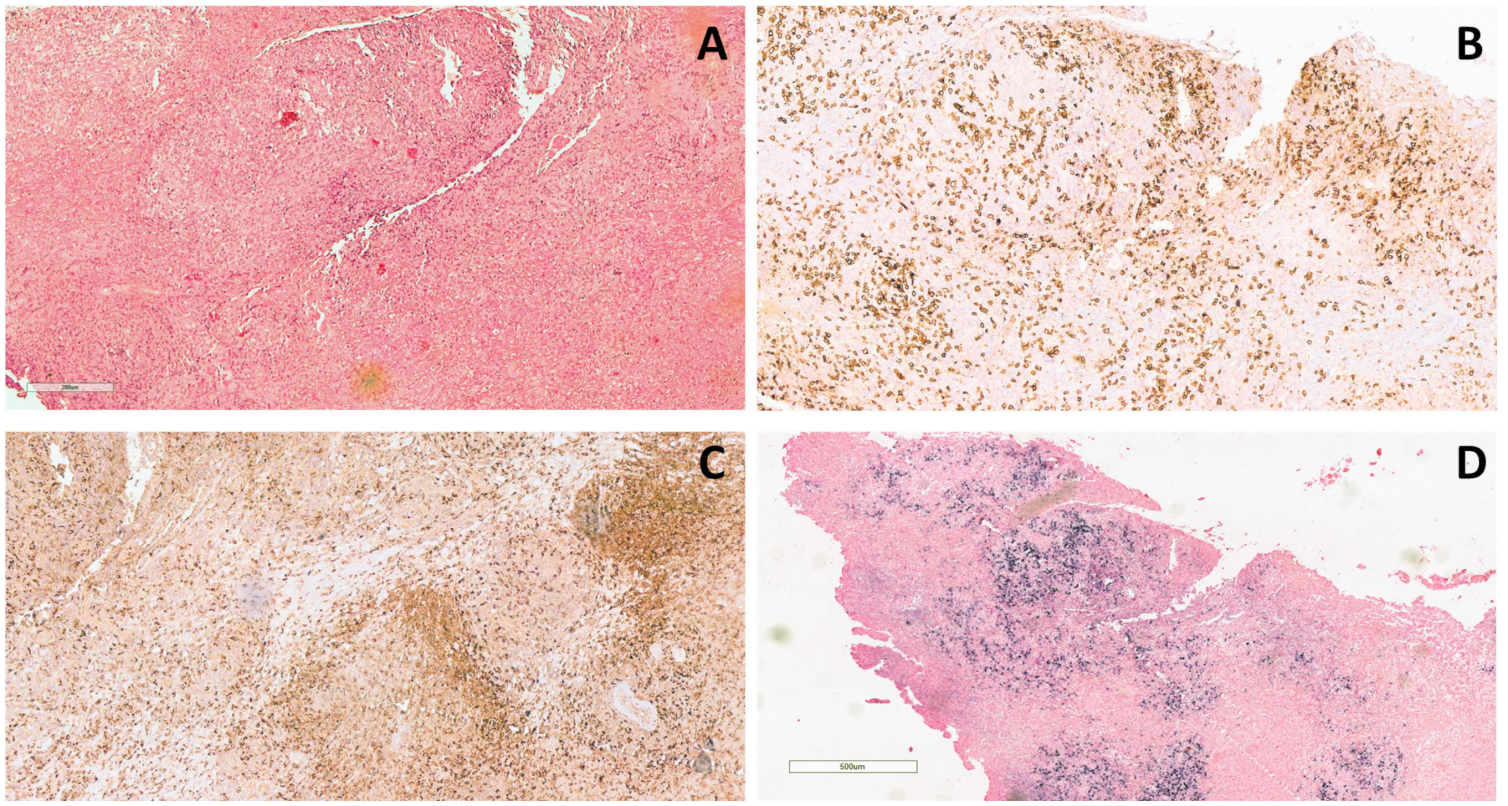
Histological examination of the lower right eyelid incisional biopsy. **(A)** Dense lymphohistiocytic infiltrate extending throughout the biopsy (hematoxylin and eosin staining, original magnification 20×). **(B)** The infiltrate is composed mainly of T-lymphocytes (CD3+) and **(C)** histiocytes (CD68+) (CD3 and CD68 immunohistochemical staining, original magnification 10×). **(D)** Positive signal upon *in situ* hybridization for EBV (EBER) (EBER immunohistochemical staining, original magnification 4×).

**Figure 3:**
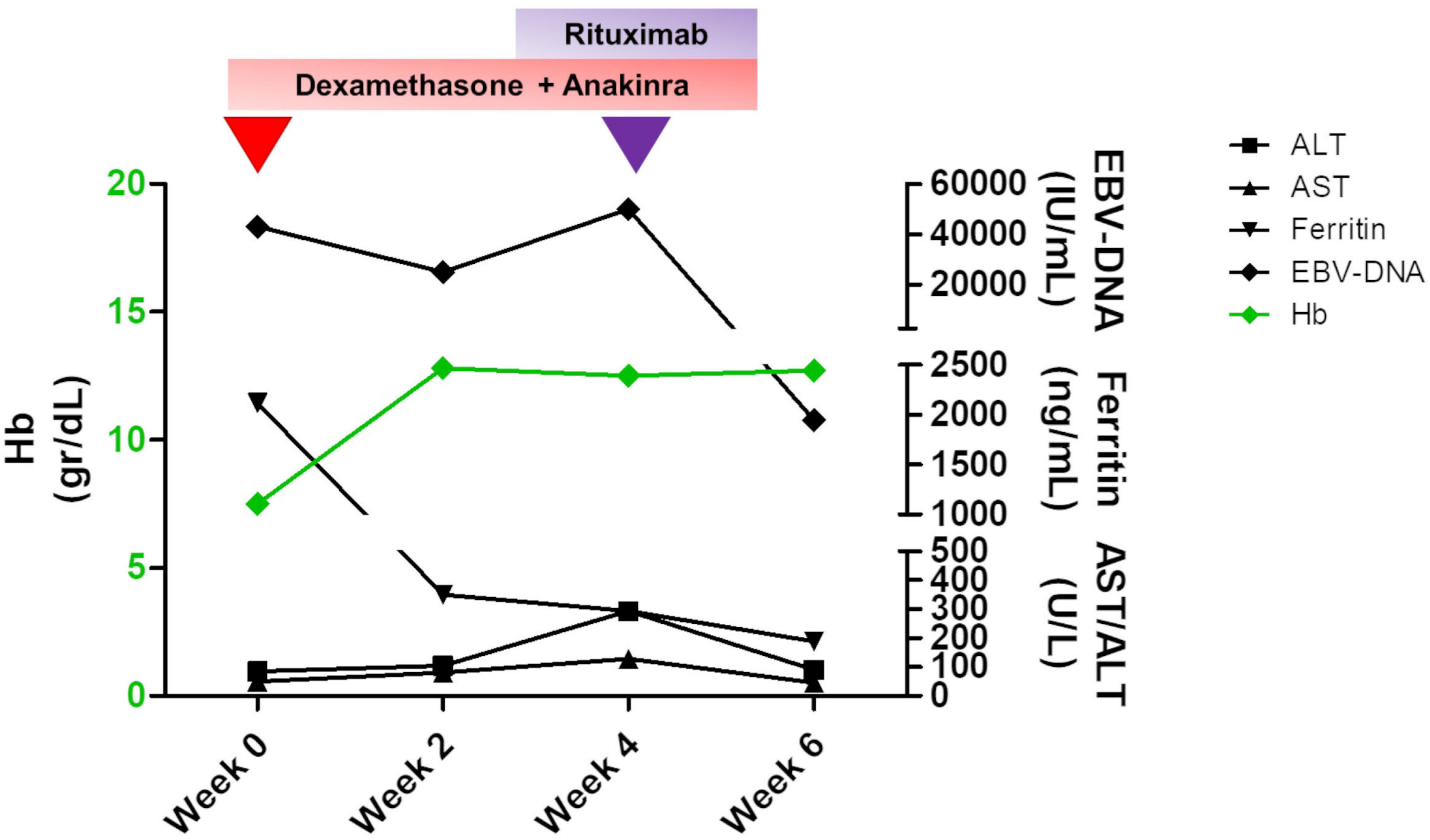
Changes in the main laboratory parameters of peripheral hemophagocytic lymphohistiocytosis (HLH) and plasma EBV-DNA levels over time. A therapy based on dexamethasone and anakinra was established to treat HLH, and an initial decrease in both biochemical values and EBV titer with an increase in hemoglobin levels was obtained by the second week of therapy. At week 4, when an increase in EBV-DNA level and persisting hypertransaminasemia were detected, rituximab was started, and a dramatic improvement was achieved. Hemoglobin levels are shown in green and are referred to the left *Y* axis. The remaining values are indicated with different symbols, as specified in the legend, and are shown on the right *Y* axis.

The bilateral lower eyelid edema (**Figure 1B**) was suggestive of pre-septal orbital cellulitis and was initially treated with local steroids and systemic antibiotics, based on the eye swab culture, positive for *Staphylococcus hominis.* However, a progressive deterioration was observed. The study of the soft facial tissues and orbits, through MRI, revealed bilateral obliteration of the ethmoid-maxillary sinuses. In both orbits, but more evident in the right one, there was an inflammatory thickening of the periorbital subcutaneous adipose tissue, hypointense in T2 (**Figure 1A**). No bony discontinuity was detected (**Figure 1A**), and this was also confirmed at the CT scan (not shown). EBV-related lymphoma was suspected, and incisional biopsy of the lower right eyelid was performed. Abnormal pinkish-brown tissue was identified below the orbicularis oculi muscles. The biopsy revealed a marked lymphohistiocytic infiltrate within the fibrous tissue, also characterized by some *foci* of necrosis and a vascular network consisting of branching vessels, with a slightly thickened wall (**Figure 2A**). Immunohistochemical studies revealed a prevalence of T lymphocytes, small and medium-sized cells with irregular nuclei, positive for CD2, CD3 (**Figure 2B**), CD5, and CD8 and a few B-lymphocytes. The staining for CD68 revealed many histiocytes (**Figure 2C**), negative for protein S100 and CD1a. TDT, CD30, pan-cytokeratin, and desmin were also negative. On *in situ* hybridization with EBER probe, many lymphocytes/macrophages were positive (**Figure 2D**). The proliferation index Ki-67 was heterogeneously expressed, with areas in which it was approximately 70% of cellularity. The morphology and immunophenotype strongly suggested an EBV-related “atypical” lymphohistiocytic proliferation. Dexamethasone at the dosage of 10 mg/m^2^, anakinra, and acyclovir were started to treat the lymphohistiocytic infiltration of the orbit and the incomplete peripheral HLH, defined based on the presence of fever, anemia, splenomegaly, and hyperferritinemia. After 2 weeks of treatment, we observed a resolution of fever and splenomegaly and a significant increase in hemoglobin and decrease in ferritin levels (**Figure 3**) and total lymphocyte count. However, after an initial decrease, EBV titer increased up to 50,000 IU/mL and hepatomegaly and hypertransaminasemia persisted under steroid therapy (**Figure 3**). Moreover, we observed a worsening of periorbital edema and nasal congestion. Rituximab was started to reduce EBV titers (6). Upon rituximab treatment, a dramatic improvement of periorbital edema and nasal congestion was observed along with a marked reduction of EBV-DNA titer (**Figure 3**). Finally, the patient underwent a successful hematopoietic stem cell transplant (HSCT).

## Review of other forms of HLH ocular infiltration

XLP1 is a heterogeneous IEI characterized by increased susceptibility to severe EBV infection and to develop HLH, dysgammaglobulinemia, and lymphoma. HLH may be triggered by viral infections, especially due to EBV or CMV (1, 6). Infiltration of activated lymphocyte and histocytes in HLH is usually detected within liver, spleen, lymph nodes, bone marrow, and central nervous system, but it can virtually affect any organ (7, 8). Lymphohistiocytic infiltration may be clinically indistinguishable from lymphomas, thus creating problems in the diagnosis and definition of the therapeutic approach especially considering that lymphoma, particularly abdominal B-cell non-Hodgkin, develops in a third of patients with XLP1. Laboratory findings and imaging may be overlapping between the two conditions and only histology plays a decisive role in the differential diagnosis. In the patient herein described, the presenting symptoms were suggestive of both HLH and lymphoma and histology helped define the nature of the infiltration driving the right therapeutic choice.

We searched PubMed for previously reported patients who developed ocular lymphohistiocytic involvement, selecting papers in which ocular signs and symptoms could be ascribed to HLH, either familial or infection-induced and excluding those in which ophthalmic manifestations were part of the pre-existing clinical picture. Posterior segment abnormalities were detected, including retinal hemorrhages requiring vitrectomy, disc edema, acute posterior multifocal placoid pigment epitheliopathy, and multiple bilateral serous pigment epithelial detachments with macular edema, resembling ocular Vogt–Koyanagi–Harada disease (9–11). Moreover, other manifestations included subconjunctival hemorrhage, ocular surface infection, orbital cellulitis, and pseudotumor (12).

## Conclusions

To our knowledge, this is the first description of periorbital involvement in HLH. In conclusion, in this case, we reported on an uncommon localization of lymphohistiocytic infiltration, which was clinically indistinguishable from lymphoma. Lymphoma-associated hemophagocytic syndrome has an extremely poor prognosis and should be promptly identified and treated to improve the outcome.

## Patient perspective

The patient and his parents were constantly informed of the diagnostic procedures, treatment options, and risks. They became aware of the rarity and complexity of the condition and agreed with its multidisciplinary management.

## Data availability statement

The raw data supporting the conclusions of this article will be made available by the authors, without undue reservation.

## Ethics statement

The studies involving humans were approved by Comitato Etico Campania 3 Sede Cardarelli. The studies were conducted in accordance with the local legislation and institutional requirements. The participants provided their written informed consent to participate in this study. Written informed consent was obtained from the participant/patient(s) for the publication of this case report.

## Author contributions

GG: Conceptualization, Writing – review & editing, Writing – original draft. VL: Writing – review & editing, Validation, Data curation. MM: Writing – review & editing, Validation. DR: Writing – review & editing. EC: Writing – review & editing. RR: Writing – original draft, Validation, Data curation. FC: Writing – review & editing. LG: Writing – review & editing. MP: Data curation, Writing – review & editing. AI: Writing – review & editing. GU: Writing – review & editing. CD: Writing – review & editing. GM: Writing – review & editing. GS: Writing – review & editing. GP: Writing – review & editing. CP: Supervision, Writing – original draft.
